# A comparison of Dutch frail older adults in 1998 and 2008: prevalences and determinants

**Published:** 2012-09-04

**Authors:** S Bremer, M.I Broese van Groenou, M Huisman, D.J.H Deeg

**Affiliations:** LASA, VU University, Amsterdam, The Netherlands; Sociology, VU University, Amsterdam, The Netherlands; Sociology VU University, EP&BS Vumc, Amsterdam, The Netherlands; Psychiatry, EP&BS Vumc, Amsterdam, The Netherlands

**Keywords:** frail older persons, prevalence, influence of socio-demographic and health features, markers

## Abstract

**Background:**

Society is facing a growing number of older people. With the increase of older people an increase in number of frail persons is expected. But will the increase in number of frail persons parallel the increase of the number of older adults? Or are the features of older adults changing in time and are these changes reducing or amplifying the increase of frail older adults?

**Objectives:**

To present the differences in prevalence of frail older persons in 1998 and in 2008 and the influence of socio-demographic and health features on the difference between these two cohorts.

**Methods:**

The data were collected in the context of the Longitudinal Aging Study Amsterdam (LASA). For this particular study we selected community dwelling respondents aged 65–88 years who had complete data in 1995 or 2008. Frailty was composed out of seven markers: BMI, cognitive functioning, grip strength, tiredness, vision and hearing problems and physical activity. Two cohorts, 1998 and 2008, were compared and the relative numbers of frail older adults in both cohorts were calculated. Next the association of different socio-demographic and health features with frailty was examined.

**Results:**

Preliminary results show a decrease in the prevalence of frail older adults from 18.5% in 1998 to 9.9% in 2008. The prevalence of frail people in 2008 for all age groups decreased compared to 1995 but in particular for the youngest age groups. In addition, the decrease in frail women is more amplified than in men. For all the markers used in defining frailty an improvement is seen for the population in 2008. Multivariate analyses will show in what degree cohort differences in frailty are related to cohort differences in education, income, co-morbidity, partner status, social network, mastery, alcohol use and smoking.

**Conclusions:**

The prevalence of frail elderly in 2008 has declined compared to 1998. This can be explained by improvements in all markers of frailty. Analyses (yet to be completed) will show the influence of socio-demographic and health features.

Powerpoint presentation available at http://www.integratedcare.org at congresses – San Marino – programme.

